# Nanoscale self-templating for oxide epitaxy with large symmetry mismatch

**DOI:** 10.1038/srep38168

**Published:** 2016-12-02

**Authors:** Xiang Gao, Shinbuhm Lee, John Nichols, Tricia L. Meyer, Thomas Z. Ward, Matthew F. Chisholm, Ho Nyung Lee

**Affiliations:** 1 Materials Science and Technology Division, Oak Ridge National Laboratory, Oak Ridge, TN 37831, USA

## Abstract

Direct observations using scanning transmission electron microscopy unveil an intriguing interfacial bi-layer that enables epitaxial growth of a strain-free, monoclinic, bronze-phase VO_2_(B) thin film on a perovskite SrTiO_3_ (STO) substrate. We observe an ultrathin (2–3 unit cells) interlayer best described as highly strained VO_2_(B) nanodomains combined with an extra (Ti,V)O_2_ layer on the TiO_2_ terminated STO (001) surface. By forming a fully coherent interface with the STO substrate and a semi-coherent interface with the strain-free epitaxial VO_2_(B) film above, the interfacial bi-layer enables the epitaxial connection of the two materials despite their large symmetry and lattice mismatch.

Epitaxial synthesis of complex oxides has stimulated considerable interest in creating novel functionalities and physical properties, where various means are used to control the close interactions among the order parameters, including lattice, spin, charge, and orbital^1^,^2^,^3^,^4^. Heterostructures of oxide materials have also played an important role in discovering novel phenomena as they can produce well-defined interfaces to couple electronic and magnetic ground states, structure, lattice, crystallographic symmetry, etc. Most studies on the epitaxial growth of complex oxides have focused on isostructural materials, e.g. perovskites on perovskites. While many binary oxides, such as TiO_2_ and VO_2_, also offer intriguing physical properties^5^,^6^,^7^,^8^,^9^,^10^,^11^, only a few substrates are available with similar structures (lattice parameters and crystal symmetry) for epitaxy thereon. Thus, gaining fundamental insight into the epitaxial growth of binary oxide thin films on lattice and symmetry mismatched substrates is of vital importance for exploring their unprecedented potential^12^,^13^,^14^.

Recently, high quality VO_2_ polymorphs were successfully stabilized as epitaxial thin films using pulsed laser epitaxy (PLE) on perovskite substrates, such as SrTiO_3_^15^,^16^,^17^. Among VO_2_ polymorphs^17^, bronze-phase VO_2_(B) has a monoclinic structure (with C2/m symmetry) whose lattice constants are *a* = 12.03, *b* = 3.69, *c* = 6.42 Å, and β = 106.6°^18^, whereas SrTiO_3_ (with Pm3m symmetry) has a cubic structure with the lattice constant of 3.905 Å. Note that while many previous studies focused on rhombohedral (R) and monoclinic (M1) phase VO_2_, recent studies in developing advanced energy storage found VO_2_(B) to be a promising cathode material for Li ion batteries^19^,^20^,^21^.

In oxide epitaxy, the important role of octahedral connectivity has been well established in perovskite-based oxide heterostructures^22^,^23^,^24^. Previous studies for heterostructures with a large symmetry mismatch observed the formation of a thin interlayer between the film and substrate induced by either phase transition^25^,^26^,^27^,^28^,^29^,^30^ or phase separation^31^,^32^,^33^. Moreover, incorporation of such an interfacial buffer layer seems to be critical, enabling the epitaxial growth between large-symmetry-mismatch materials^34^,^35^,^36^,^37^,^38^. Interestingly, a previous study based on a STEM observation reported the formation of an imperfect structure at a VO_2_(B)/SrTiO_3_ interface^16^. However, details about the interface structure have not been explored.

In this work, we report how two very dissimilar materials can form an epitaxial heterostructure by aberration-corrected scanning transmission electron microscopy (STEM) imaging and electron energy-loss spectroscopy (EELS). We have found an interfacial bi-layer at the VO_2_(B)/STO interface that enables epitaxial growth of a structurally complex, low symmetry film on a high symmetry substrate. It is rather surprising that VO_2_(B) films with corner- and edge-sharing oxygen octahedra (see Fig. 1a and b) can be epitaxially grown on STO with corner-sharing octahedra, despite the different oxygen networks and the large biaxial lattice mismatch.

## Results and Discussion

**Figure 1:**
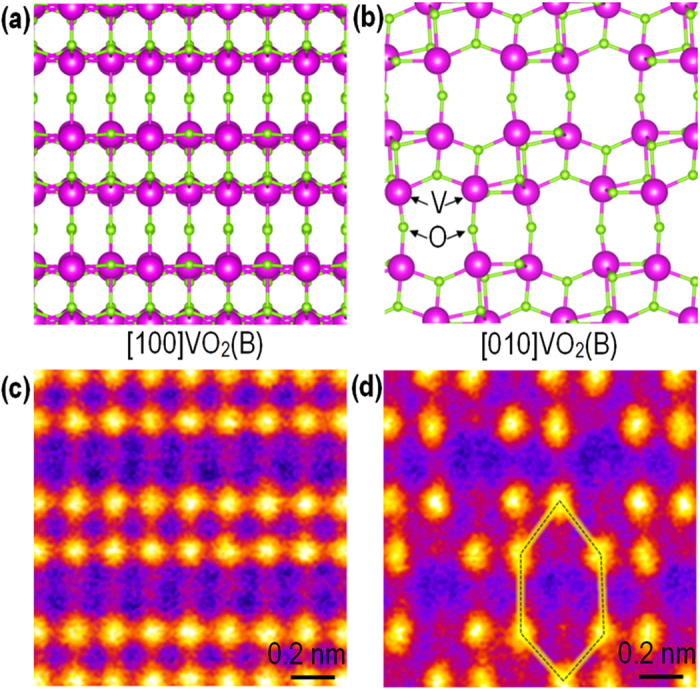
Atomic structure of VO_2_(B). (**a,b**) Schematics and (**c,d**) corresponding cross-sectional HAADF images of VO_2_(B) seen along (**a,c**) the [100] and (**b,d**) [010] directions. The hexagon in (**d**) indicates the large open channel in VO_2_(B) useful for ionic conduction.

High quality VO_2_(B) epitaxial films were grown on (001)-oriented STO by PLE under well-optimized growth conditions. The details on the epitaxial growth and crystal quality as well as associated physical properties can be found elsewhere^17^. Figure 1 shows atomic structure projections and corresponding cross-sectional high-angle annular dark-field (HAADF) images taken along the [100]_VO2(B)_ and [010]_VO2(B)_ directions of VO_2_(B). In the *Z*-contrast HAADF images, the cation columns containing Ti (Z = 22), V (Z = 23), and Sr (Z = 38) are seen with intensities strongly dependent on their atomic number, while columns containing only light O (Z = 8) atoms are hardly visible. The image shown in Figure 1d provides the reason why VO_2_(B) is of particular interest for energy storage as the atomic structure seen from the [010]_VO2(B)_ direction features an open framework that offers a good ionic diffusion pathway. The structural projection along [010]_VO2(B)_ also reveals the clear symmetry mismatch between the film and substrate. Thus, we chose this orientation for the majority of the STEM investigations.

**Figure 2:**
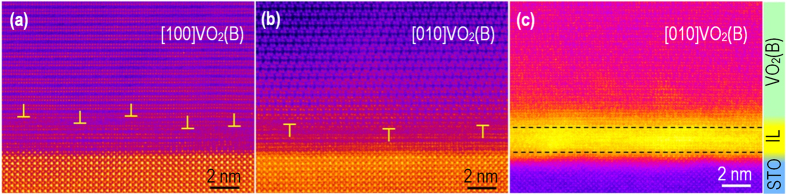
Microstructure of the VO_2_(B)/STO interface. HAADF images show two growth twins orthogonally oriented along (**a**) the [100]_VO2(B)_ and (**b**) [010]_VO2(B)_ directions with respect to the [100]_STO_ direction. (**c**) LAADF image taken from the image in (**b**), showing an extra intensity from the interfacial layer (IL) associated with increased electron beam dechanneling and, thus, scattering of electrons due to increased atomic disorder.

Figures 2a and b show cross-sectional HAADF images of an epitaxial VO_2_(B) film grown on a STO substrate. The images were taken along the [100]_STO_ direction. As shown in Figures 2a and b, the film is found to contain at least two domains aligned parallel to the [100]_VO2(B)_ and [010]_VO2(B)_ directions, i.e. orthogonally positioned with respect to the [100] direction of the STO substrate. In fact, the film contains two additional domains that are rotated 180 degrees about the surface normal from those imaged in Fig. 2. A thin (typically ~2 nm thick) interfacial layer (IL) can be seen between the VO_2_(B) film and the STO substrate. Based on fast-Fourier transformation (FFT) analysis, an array of misfit dislocations has formed between the IL and the structurally relaxed VO_2_(B) film (as indicated in Fig. 2a and b, and in the corresponding FFT images in Figure S1 in Supporting Information). The interface between the STO substrate and the IL appears to be fully coherent. As shown in Figure 2a and b, the average spacing between dislocations observed along the [100]_VO2(B)_ direction is 3.6 ± 0.9 nm, while it is 7.9 ± 1.1 nm when seen along the [010]_VO2(B)_ direction. These spacings are in good agreement with calculated values of ~3.5 nm and ~7.2 nm obtained using the lattice mismatch of +5.5% for the [100]VO_2_(B) || [100]STO projection and −2.7% for the orthogonal [010]VO_2_(B)||[100]STO projection. This result reveals unambiguously that the large bi-axial lattice mismatch between the film and substrate is accommodated by the creation of dislocations at the VO_2_(B)/TL interface, i.e. strain-free VO_2_(B) epitaxial films are obtained.

Figure 2c shows a low-angle annular dark-field (LAADF) image taken from the sample seen along the [010]_VO2(B)_/[100]_STO_ direction. The LAADF image highlights the interlayer, which is substantially brighter than the film or the substrate. This brighter contrast in a LAADF image indicates that the IL has a higher level of structural disorder, which leads to the electron dechannelling of the incident beam^39^,^40^,^41^. Based on a geometric phase analysis (GPA), it is also seen that the VO_2_ in the IL undergoes a significant lattice expansion along the film surface normal as compared to the VO_2_(B) film (see Figure S2 in Supporting Information). This result is consistent with the rather large in-plane compression (−5.5%) of the VO_2_(B) film in the [100]_VO2(B)_/[100]_STO_ projection that will cause the observed out-of-plane expansion.

**Figure 3:**
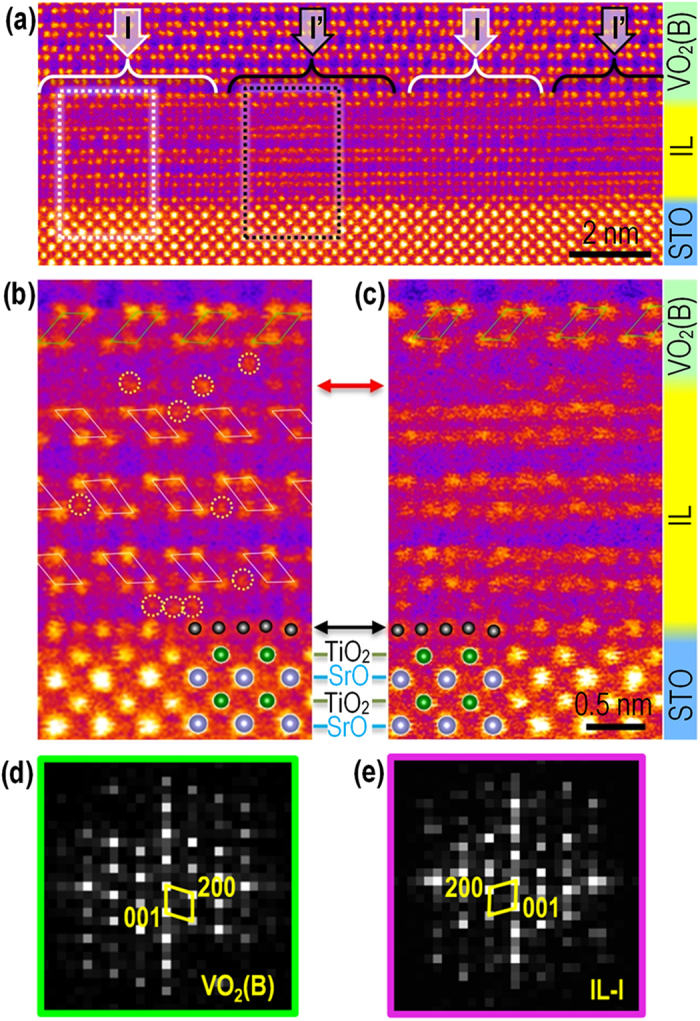
High-resolution observation of interfacial layer (IL). (**a**) HAADF image showing that the IL consists of nanodomains, e.g. I and I′. (**b,c**) Magnified HAADF images taken from nanodomains I and I′ marked by the dashed rectangles in a) showing in greater detail the local atom arrangements. (**d,e**) FFT electron diffraction patterns obtained from the VO_2_(B) film and the IL (nanodomain-I), respectively. The red and black arrows between (**b,c**) indicate extra atom planes formed at the upper and lower sides of IL, respectively.

Figure 3a shows a HAADF image of the IL taken along the [010]_VO2(B)_ direction. While there is a region of the IL (Fig. 3b) that clearly duplicates the projected structure of the relaxed VO_2_(B) film above it (except that it is rotated 180 degrees about the surface normal), most of this layer (and its FFT, Fig. 3d,e) looks to be a superposition of [100], [−100], [010], and [0–10] projections of epitaxially strained VO_2_(B). The other important feature of the IL is the extra atomic layer between the STO and VO_2_(B) indicated with a black arrow in Fig. 3b and c. The atomic layer shows a periodic, but different arrangement of B-site atoms than that of the TiO_2_-terminated STO surface. The HAADF images show the out-of-plane lattice spacing between the topmost TiO_2_ layer of STO and the extra (Ti,V)-O layer to be 2.4 ± 0.1 Å, which is significantly larger than the 1.9 Å (001) plane spacing in STO. The intensity variations indicate that, in this [100]_STO_ projection, the extra layer contains additional (Ti,V) columns with roughly 1/2 the B-site density of its neighboring atom columns.

**Figure 4:**
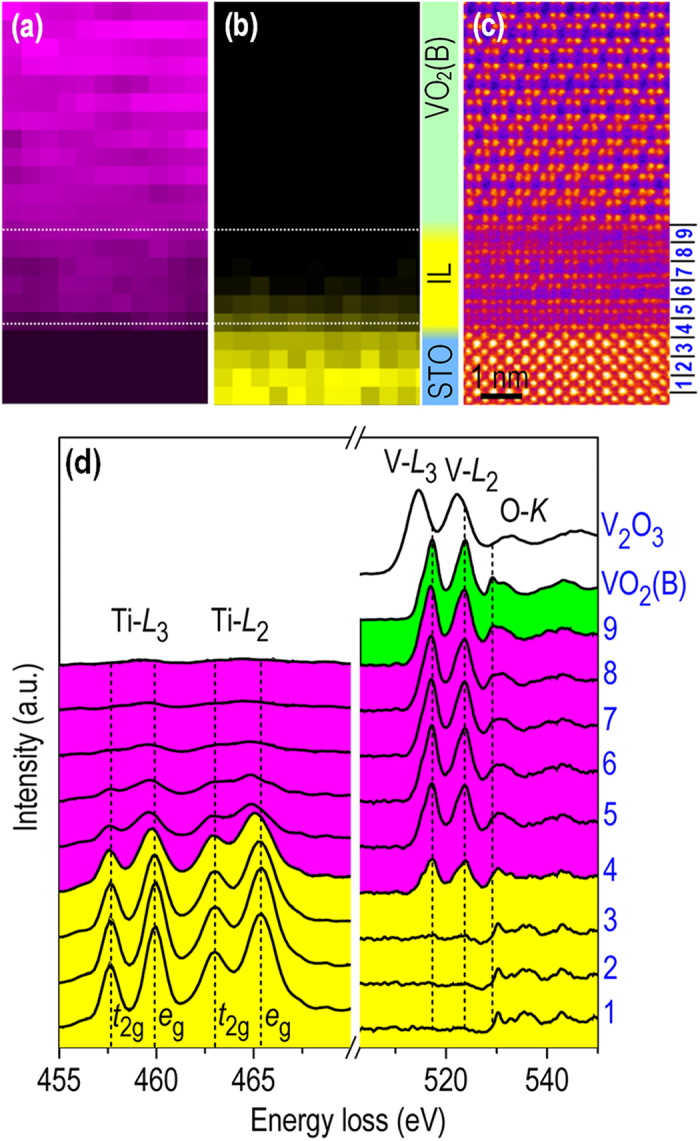
Layer-by-layer EELS analysis. Elemental maps for (**a**) V-*L*_2,3_ and (**b**) Ti-*L*_2,3_ signals obtained from the interface region shown in (**c**). (**d**) Back-ground subtracted Ti-*L*_2,3_, V-*L*_2,3_, and O-*K* spectra obtained across the interface. The EELS spectra numbered 1 through 9 are obtained from the local atomic planes indicated in (**c**). EELS profile intensity is normalized using the O-*K* edge beyond the ionization transitions to discrete states.

Spatially resolved STEM-EELS data from the interfacial region is presented in Fig. 4. Figure 4a and b respectively show element maps using the Ti-*L*_2,3_ and V-*L*_2,3_ signals taken from the same interfacial region shown in Fig. 4c. The V-*L*_2,3_ signal in Fig. 4a shows a chemically abrupt interface between the film and STO substrate. On the other hand, the Ti-*L*_2,3_ signal is seen to extend into the IL. Figure 4d shows background-subtracted Ti-*L*, V-*L* and O-*K* EELS profiles obtained layer-by-layer across the IL. Standard spectra obtained from single-crystalline VO_2_(B) (V^4+^) and V_2_O_3_ (V^3+^) thin films are also included for comparison. The peak position of V-*L*_*2,3*_ edges are seen to remain fixed indicating little to no change in the valence state of V in the IL and the VO_2_(B) film.

The Ti-*L*_2,3_ EELS fine structure obtained from the extra (Ti,V)-O layer on the STO substrate surface shows broadened *L*_3_ and *L*_2_ edges, as well as a shift of the *e*_*g*_ peaks toward lower energy-loss (see Fig. 4d). The observed electronic state and atomic structure of this extra layer are in good agreement with previous theoretical simulations^42^ and STEM observations^43^ of a c (4 × 2) reconstructed STO (001) surface composed of a double-layer TiO_2_. The topmost layer of this reconstructed surface was predicted to contain clustered quartets of edge-sharing square-pyramidal TiO_5_^42^,^43^. The extra (Ti,V)-O layer on TiO_2_-terminated STO can introduce edge sharing oxygen containing units, which are more consistent with the VO_2_(B) structure. To our knowledge, the formation of such an interface bi-layer can not be explained by any existing growth model that involves either phase transition^25^,^26^,^27^,^28^,^29^,^30^ or phase separation^31^,^32^,^33^ at film/substrate interfaces to accommodate inter-phase structural discontinuities.

The observed results reveal unambiguously, at the initial growth stage, the formation of an interfacial layer composed of VO_2_(B) nanodomains that enable the epitaxy of VO_2_(B) on STO. This epitaxy with a large symmetry mismatch involves a structural reconstruction process at the substrate surface to facilitate the symmetry transition between the two distinct component structures. The VO_2_(B) nanodomains form a fully coherent interface with the STO substrate and are subject to considerable lattice strain. Once the strain energy in the VO_2_(B) nanodomains exceeds a critical level, misfit dislocations are introduced, followed by the growth of fully relaxed VO_2_(B). The much larger domain size in the relatively strain-free film is an expected result of increased adatom mobility on the relaxed surface. Formation of the interfacial VO_2_(B) nanodomains indicates a nanoscale self-templating process that enables the epitaxy of strain-free VO_2_(B) films on STO substrates. Therefore, the results not only enable novel insights into atomic mechanism of complex heterostructure interface at an atomic scale, but also shed light on the epitaxial design of two materials with large symmetry and lattice mismatch.

## Methods

### Epitaxial synthesis

VO_2_(B) epitaxial films were deposited on (001) SrTiO_3_ substrates by pulsed laser epitaxy. A sintered ceramic VO_2_ target was ablated with a KrF excimer laser (λ = 248 nm) at a repetition rate of 5 Hz and laser fluence of 1 J·cm^−2^. The optimized substrate temperature and oxygen pressure to grow high quality thin films were 500 °C and 20 mTorr, respectively, and the samples were *in-*situ post-annealed in 1 atm of O_2_ for 1 hour at the growth temperature to ensure the oxygen stoichiometry. Detailed information on the synthesis of single-crystalline VO_2_(B) (V^4+^) and V_2_O_3_ (V^3+^) thin films utilized for EELS analysis can be found elsewhere^14^.

### Scanning Transmission Electron Microscopy (STEM)

Cross-sectional specimens oriented along the [100] STO direction for STEM analysis were prepared using ion milling after mechanical thinning and precision polishing (using water-free abrasive). High-angle annular dark-field (HAADF) and low-angle annular dark-field (LAADF) imaging and electron-energy loss spectroscopy (EELS) analysis were carried out in Nion UltraSTEM200 operated at 200 keV. The microscope is equipped with a cold field-emission gun and a corrector of third- and fifth-order aberrations for sub-angstrom resolution. Inner/outer detector angles of 78/240 mrad and 30/63 mrad were used for HAADF and LAADF imaging, respectively. The convergence semi-angle for the electron probe was set to 30 mrad.

## Additional Information

**How to cite this article**: Gao, X. *et al*. Nanoscale self-templating for oxide epitaxy with large symmetry mismatch. *Sci. Rep.* **6**, 38168; doi: 10.1038/srep38168 (2016).

**Publisher's note:** Springer Nature remains neutral with regard to jurisdictional claims in published maps and institutional affiliations.

## Supplementary Material

Supplementary Information

## References

[b1] Hwang H. Y. . Emergent phenomena at oxide interfaces. Nature materials 11, 103–113 (2012). 22270825 10.1038/nmat3223

[b2] Zubko P., Gariglio S., Gabay M., Ghosez P. & Triscone J.-M. Interface physics in complex oxide heterostructures. Annu. Rev. Condens. Matter Phys. 2, 141–165 (2011).

[b3] Jeen H. . Reversible redox reactions in an epitaxially stabilized SrCoOx oxygen sponge. Nature materials 12, 1057–1063 (2013). 23975056 10.1038/nmat3736

[b4] Gorbenko O. Y., Samoilenkov S., Graboy I. & Kaul A. Epitaxial stabilization of oxides in thin films. Chemistry of materials 14, 4026–4043 (2002).

[b5] Morin F. Oxides which show a metal-to-insulator transition at the Neel temperature. Physical Review Letters 3, 34 (1959).

[b6] O’regan B. & Grfitzeli M. A low-cost, high-efficiency solar cell based on dye-sensitized. nature 353, 737–740 (1991).

[b7] Arico A. S., Bruce P., Scrosati B., Tarascon J.-M. & Van Schalkwijk W. Nanostructured materials for advanced energy conversion and storage devices. Nature materials 4, 366–377 (2005). 15867920 10.1038/nmat1368

[b8] Khan S. U., Al-Shahry M. & Ingler W. B. Efficient photochemical water splitting by a chemically modified n-TiO_2_. science 297, 2243–2245 (2002). 12351783 10.1126/science.1075035

[b9] Yang Z., Ko C. & Ramanathan S. Oxide electronics utilizing ultrafast metal-insulator transitions. Annual Review of Materials Research 41, 337–367 (2011).

[b10] Park J. H. . Measurement of a solid-state triple point at the metal-insulator transition in VO_2_. nature 500, 431–434 (2013). 23969461 10.1038/nature12425

[b11] Morrison V. R. . A photoinduced metal-like phase of monoclinic VO_2_ revealed by ultrafast electron diffraction. science 346, 445–448 (2014). 25342797 10.1126/science.1253779

[b12] Jeong J. . Suppression of metal-insulator transition in VO_2_ by electric field–induced oxygen vacancy formation. science 339, 1402–1405 (2013). 23520104 10.1126/science.1230512

[b13] Luttrell T. . Why is anatase a better photocatalyst than rutile?-Model studies on epitaxial TiO_2_ films. Scientific reports 4 (2014). 10.1038/srep04043 PMC3918909 24509651

[b14] Lee S., Meyer T. L., Park S., Egami T. & Lee H. N. Growth control of the oxidation state in vanadium oxide thin films. Applied Physics Letters 105, 223515 (2014).

[b15] Chen A. . Textured metastable VO_2_(B) thin films on SrTiO_3_ substrates with significantly enhanced conductivity. Applied Physics Letters 104, 071909 (2014).

[b16] Srivastava A. . Selective growth of single phase VO_2_ (A, B, and M) polymorph thin films. APL materials 3, 026101 (2015).

[b17] Lee S., Ivanov I. N., Keum J. K. & Lee H. N. Epitaxial stabilization and phase instability of VO_2_ polymorphs. Scientific reports 6 (2016). 10.1038/srep19621 PMC4726436 26787259

[b18] Pokrovskii B. & Khachaturyan A. The concentration wave approach to the pairwise interaction model for predicting the crystal structures of ceramics, I. Journal of Solid State Chemistry 61, 137–153 (1986).

[b19] Li W., Dahn J. R. & Wainwright D. S. Rechargeable lithium batteries with aqueous electrolytes. Science-AAAS-Weekly Paper Edition-including Guide to Scientific Information 264, 1115–1117 (1994). 10.1126/science.264.5162.1115 17744893

[b20] Mai L. . Nanoscroll Buffered Hybrid Nanostructural VO_2_(B) Cathodes for High‐Rate and Long-Life Lithium Storage. Advanced materials 25, 2969–2973 (2013). 23519912 10.1002/adma.201205185

[b21] Niu C. . VO_2_ nanowires assembled into hollow microspheres for high-rate and long-life lithium batteries. Nano letters 14, 2873–2878 (2014). 24742281 10.1021/nl500915b

[b22] Rondinelli J. M., May S. J. & Freeland J. W. Control of octahedral connectivity in perovskite oxide heterostructures: An emerging route to multifunctional materials discovery. MRS bulletin 37, 261–270 (2012).

[b23] Meyer T. L. . Symmetry-Driven Atomic Rearrangement at a Brownmillerite–Perovskite Interface. Advanced Electronic Materials 2 (2016).

[b24] Kim T. . Polar metals by geometric design. Nature 533, 68–72 (2016). 27096369 10.1038/nature17628

[b25] Lazarides N., Paltoglou V., Maniadis P., Tsironis G. & Panagopoulos C. Strain-induced interface reconstruction in epitaxial heterostructures. Physical Review B 84, 245428 (2011).

[b26] Zhou H., Chisholm M. F., Yang T.-H., Pennycook S. J. & Narayan J. Role of interfacial transition layers in VO_2_/Al_2_O_3_ heterostructures. Journal of Applied Physics 110, 073515 (2011).

[b27] Bayati M. . Domain epitaxy in TiO_2/α_-Al_2_O_3_ thin film heterostructures with Ti_2_O_3_ transient layer. Applied Physics Letters 100, 251606 (2012).

[b28] Chen A. . A New Class of Room-Temperature Multiferroic Thin Films with Bismuth‐Based Supercell Structure. Advanced Materials 25, 1028–1032 (2013). 23180693 10.1002/adma.201203051

[b29] Zhu Y. . Research Updates: Epitaxial strain relaxation and associated interfacial reconstructions: The driving force for creating new structures with integrated functionality. APL materials 1, 050702 (2013).

[b30] Pennycook S. . Misfit accommodation in oxide thin film heterostructures. Acta Materialia 61, 2725–2733 (2013).

[b31] Lazarov V. K., Chambers S. A. & Gajdardziska-Josifovska M. Polar oxide interface stabilization by formation of metallic nanocrystals. Physical Review Letters 90, 216108 (2003). 12786572 10.1103/PhysRevLett.90.216108

[b32] Turner S. . Structural phase transition and spontaneous interface reconstruction in La_2/3_Ca_1/3_MnO_3_/BaTiO_3_ superlattices. Physical Review B 87, 035418 (2013).

[b33] Gao X. . Structural Distortion and Compositional Gradients Adjacent to Epitaxial LiMn_2_O_4_ Thin Film Interfaces. Advanced Materials Interfaces 1 (2014).

[b34] Li L. . Strain and Interface Effects in a Novel Bismuth-Based Self-Assembled Supercell Structure. ACS applied materials & interfaces 7, 11631–11636 (2015). 25951914 10.1021/acsami.5b02699

[b35] Zhang K. . Water-Free Titania-Bronze Thin Films with Superfast Lithium‐Ion Transport. Advanced Materials 26, 7365–7370 (2014). 25244308 10.1002/adma.201401757

[b36] Lee D. . Oxygen surface exchange kinetics and stability of (La, Sr)_2_CoO_4±δ_/La_1−x_Sr_x_MO_3−δ_ (M= Co and Fe) hetero-interfaces at intermediate temperatures. Journal of Materials Chemistry A 3, 2144–2157 (2015).

[b37] Lee D. . Strontium influence on the oxygen electrocatalysis of La_2−x_Sr_x_NiO_4±δ_ (0.0 ≤ x_Sr_ ≤ 1.0) thin films. Journal of Materials Chemistry A 2, 6480–6487 (2014).

[b38] Lee D. . Enhanced Oxygen Surface Exchange Kinetics and Stability on Epitaxial La_0.8_Sr_0. 2_CoO_3−δ_ Thin Films by La_0.8_Sr_0. 2_MnO_3−δ_ Decoration. The Journal of Physical Chemistry C 118, 14326–14334 (2014).

[b39] Cowley J. & Huang Y. De-channelling contrast in annular dark-field STEM. Ultramicroscopy 40, 171–180 (1992).

[b40] Hillyard S. & Silcox J. Detector geometry, thermal diffuse scattering and strain effects in ADF STEM imaging. Ultramicroscopy 58, 6–17 (1995).

[b41] Pennycook S. J. & Nellist P. D. Scanning transmission electron microscopy: imaging and analysis. (Springer Science & Business Media, 2011).

[b42] Erdman N. . Surface structures of SrTiO_3_ (001): A TiO_2_-rich reconstruction with ac (4 × 2) unit cell. Journal of the American Chemical Society 125, 10050–10056 (2003). 12914468 10.1021/ja034933h

[b43] Zhu G.-z., Radtke G. & Botton G. A. Bonding and structure of a reconstructed (001) surface of SrTiO_3_ from TEM. nature 490, 384–387 (2012). 23051749 10.1038/nature11563

