# Supplementary material for: Nanoscale self-templating for oxide epitaxy with large symmetry mismatch
Source: Sci Rep. 2016 Dec 2;6:38168. doi: 10.1038/srep38168 (PMC5133589; doi:10.1038/srep38168)
Supplement: Supplementary Information [file srep38168-s1.pdf]

Supplementary Information

Nanoscale self-templating for oxide epitaxy with large symmetry mismatch

Xiang Gao, Shinbuhm Lee, John Nichols, Tricia L. Meyer, Thomas Z. Ward,

Matthew F. Chisholm, and Ho Nyung Lee*

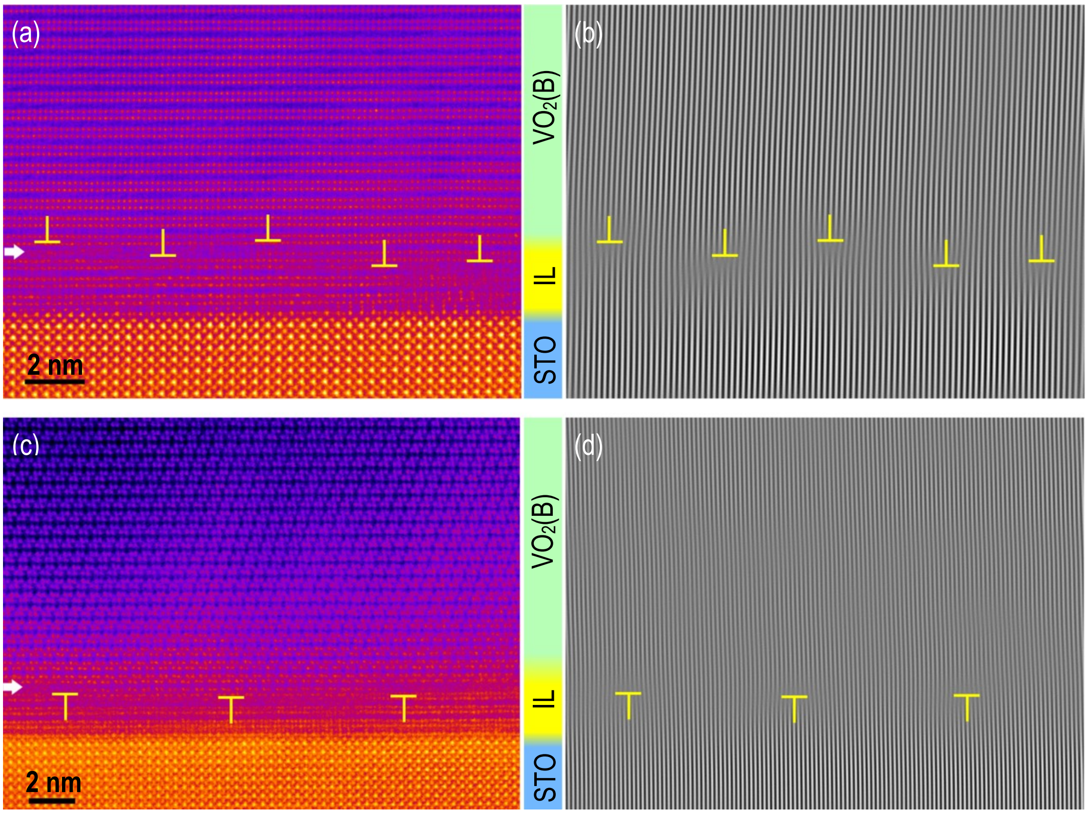

Figure S1. Formation of periodical dislocations and stacking faults at the VO_2(B)/IL interface. a,b) HAADF images taken along the [100]- and [010]-oriented VO_2(B) domains and c,d) their FFT maps, respectively. White arrows in a) and b) indicate the formation of stacking faults at the VO_2(B)/IL interface, which should account for the observation of wide-dark gaps displaying blurry atom contrasts (as arrowed) in the HAADF images.

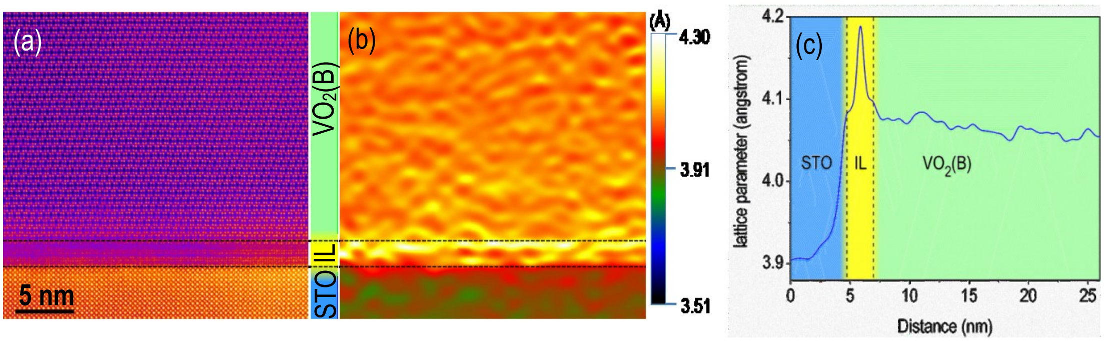

Figure S2. Geometric phase analysis (GPA). a) HAADF image taken along the [010]VO_2(B)/[100]STO direction. b) Map of out-of-plane lattice parameter (normalized to the STO bulk lattice parameter of 0.3905 nm) of a). c) Integrated out-of-plane lattice parameter profile from b) as a function of distance. The results reveal unambiguously the out-of-plane lattice expansion in the IL compared to the VO_2(B) film. By forming a fully coherent interface with the STO substrate, the IL adopts an in-plane unit size of 0.3905×0.3905 nm^2, which is larger than the corresponding value of $(1.203/3)\times0.369$ nm^2 of the VO_2(B). Therefore the IL undergoes a significant lattice expansion as compared to the VO_2(B) film.
